# Prognostic impact of *PDGFRA* gain/amplification and *MGMT* promoter methylation status in patients with *IDH* wild-type glioblastoma

**DOI:** 10.1093/noajnl/vdac097

**Published:** 2022-06-21

**Authors:** Nayuta Higa, Toshiaki Akahane, Seiya Yokoyama, Hajime Yonezawa, Hiroyuki Uchida, Tomoko Takajo, Ryosuke Otsuji, Taiji Hamada, Kei Matsuo, Mari Kirishima, Nobuhiro Hata, Ryosuke Hanaya, Akihide Tanimoto, Koji Yoshimoto

**Affiliations:** Department of Neurosurgery, Graduate School of Medical and Dental Sciences, Kagoshima University, Kagoshima-City, Kagoshima, Japan; Department of Pathology, Graduate School of Medical and Dental Sciences, Kagoshima University, Kagoshima-City, Kagoshima, Japan; Center for Human Genome and Gene Analysis, Kagoshima University Hospital, Kagoshima-City, Kagoshima, Japan; Department of Pathology, Graduate School of Medical and Dental Sciences, Kagoshima University, Kagoshima-City, Kagoshima, Japan; Department of Neurosurgery, Graduate School of Medical and Dental Sciences, Kagoshima University, Kagoshima-City, Kagoshima, Japan; Department of Neurosurgery, Graduate School of Medical and Dental Sciences, Kagoshima University, Kagoshima-City, Kagoshima, Japan; Department of Neurosurgery, Graduate School of Medical and Dental Sciences, Kagoshima University, Kagoshima-City, Kagoshima, Japan; Department of Neurosurgery, Graduate School of Medical Sciences, Kyushu University, Fukuoka, Japan; Department of Pathology, Graduate School of Medical and Dental Sciences, Kagoshima University, Kagoshima-City, Kagoshima, Japan; Department of Pathology, Graduate School of Medical and Dental Sciences, Kagoshima University, Kagoshima-City, Kagoshima, Japan; Department of Pathology, Graduate School of Medical and Dental Sciences, Kagoshima University, Kagoshima-City, Kagoshima, Japan; Department of Neurosurgery, Graduate School of Medical Sciences, Kyushu University, Fukuoka, Japan; Department of Neurosurgery, Graduate School of Medical and Dental Sciences, Kagoshima University, Kagoshima-City, Kagoshima, Japan; Department of Pathology, Graduate School of Medical and Dental Sciences, Kagoshima University, Kagoshima-City, Kagoshima, Japan; Center for Human Genome and Gene Analysis, Kagoshima University Hospital, Kagoshima-City, Kagoshima, Japan; Department of Neurosurgery, Graduate School of Medical and Dental Sciences, Kagoshima University, Kagoshima-City, Kagoshima, Japan; Department of Neurosurgery, Graduate School of Medical Sciences, Kyushu University, Fukuoka, Japan

**Keywords:** glioblastoma, *IDH* wild-type, *MGMTp*, *PDGFRA* gain/ amplification, prognostic markers

## Abstract

**Background:**

Platelet-derived growth factor receptor alpha (*PDGFRA*) is the second most frequently mutated tyrosine kinase receptor in glioblastoma (GBM). However, the prognostic impact of *PDGFRA* amplification on GBM patients remains unclear. Herein, we evaluated this impact by retrospectively analyzing outcomes of patients with *IDH* wild-type GBM.

**Methods:**

Using a custom-made oncopanel, we evaluated *PDGFRA* gain/amplification in 107 GBM samples harboring wild-type *IDH*, along with *MGMT* promoter (*MGMTp*) methylation status.

**Results:**

We detected *PDGFRA* gain/amplification in 31 samples (29.0%). *PDGFRA* gain/amplification predicted poor prognosis (*P* = .003). Compared to unamplified *PDGFRA*, *PDGFRA* gain/amplification in GBM was associated with higher patient age (*P* = .031), higher Ki-67 score (*P* = .019), and lower extent of surgical resection (*P* = .033). Unmethylated *MGMTp* also predicted poor prognosis (*P* = .005). As *PDGFRA* gain/amplification and unmethylated *MGMTp* were independent factors for poor prognosis in multivariate analyses, we grouped GBM cases based on *PDGFRA* and *MGMTp* status: poor (*PDGFRA* gain/amplification and unmethylated *MGMTp*), intermediate (*PDGFRA* gain/amplification or unmethylated *MGMTp*), and good (*PDGFRA* intact and methylated *MGMTp*) prognosis. The Kaplan-Meier survival analysis indicated that these groups significantly correlated with the OS of GBM patients (*P* < .001).

**Conclusions:**

Here we report that *PDGFRA* gain/amplification is a predictor of poor prognosis in *IDH* wild-type GBM. Combining *PDGFRA* gain/amplification with *MGMTp* methylation status improves individual prognosis prediction in patients with *IDH* wild-type GBM.

**Key Points**

- The median OS varies between GBM patients with and without *PDGFRA* gain/amplification.
- *PDGFRA* and *MGMTp* statuses determine patient prognoses in GBM.

**Importance of the Study**

Recently, it has been reported that *IDH* wild-type glioblastoma has different molecular subgroups that have distinct clinical features and prognoses. Although *PDGFRA* is the second most frequently mutated tyrosine kinase receptor in glioblastoma, the prognostic impact of its gain/amplification in glioblastoma patients remains unclear. Here, we demonstrated that *PDGFRA* gain/amplification is associated with poor prognosis in *IDH* wild-type glioblastoma. Moreover, using multivariate analysis, we determined that *PDGFRA* gain/amplification and *MGMTp* methylation status were independent prognostic markers. We hypothesized that these markers could improve the risk stratification of *IDH* wild-type glioblastoma. Additionally, we determined that glioblastomas could be subdivided into 3 groups based on the status of *PDGFRA* and *MGMTp*: poor (both *PDGFRA* gain/amplification and unmethylated *MGMTp*), intermediate (either *PDGFRA* gain/amplification or unmethylated *MGMTp*), and good (both *PDGFRA* intact and methylated *MGMTp*) prognosis groups. Such stratification will likely provide precise information to patients and can help influence their bedside decisions.

The gene encoding platelet-derived growth factor receptor alpha (*PDGFRA*) is present on chromosome 4q12. *PDGFRA* is the second most frequently mutated tyrosine kinase receptor-encoding gene, following *EGFR*, in glioblastoma (GBM)^1,2^ and plays an important role in oligodendrocyte differentiation.^3^ However, amplification of *PDGFRA* is associated with oligodendroglial morphology and malignancy.^4^ PDGFRA is a transmembrane receptor comprising 5 immunoglobulin-like extracellular domains and an intracellular tyrosine kinase domain. The PDGFR signaling pathway activates intracellular signaling pathways, such as the RAS/MAPK and PI3K/AKT pathways, that are involved in cell proliferation, migration, survival, and oncogenesis.^5,6^ Furthermore, the concept of PDGFRA as a possible drug target for GBM has been gaining attention. In fact, several PDGFRA-targeting antitumor agents, such as imatinib, sorafenib, nilotinib, and sunitinib, have already been developed.^7^ In the clinical scenario, pediatric GBM cases have a higher incidence of *PDGFRA* amplification than adult cases,^8^ and *PDGFRA* amplification is associated with the involvement of carpus callosum in GBM.^9,10^ In addition, it has been associated with poor patient survival in diffuse *H3K27M*-mutant midline gliomas.^11^ However, the prognostic value of *PDGFRA* amplification in GBM remains controversial, despite its relatively high frequency of occurrence in patients with GBM.

In this study, we aimed to identify the potential clinically distinct subgroups of *IDH* wild-type GBMs by examining the correlation between *PDGFRA* gain/amplification and patient survival.

## Materials and Methods

### Glioblastoma Samples

We obtained 107 tumor tissue samples from the Central Nervous System Tumor Tissue Bank of the Kagoshima University Hospital. These samples corresponded to 107 patients with GBM with a mean age of 67 years: 61 men (57.0%) and 46 women (43.0%). This study was approved by the Institutional Review Board of Kagoshima University (approval number: 180104) and complied with the principles of the Declaration of Helsinki. Informed consent was obtained from all patients. All 107 tumor samples were grade 4 *IDH* wild-type GBMs and were classified according to the 2021 World Health Organization classification of tumors of the central nervous system. We prepared formalin-fixed paraffin-embedded (FFPE) tumor samples by fixing the resected tumors with phosphate-buffered 10% formalin within 24 hours of sampling. Consecutively, we routinely processed them for paraffin embedding and sectioned them for hematoxylin and eosin staining. All the tissues were histologically evaluated by board-certified pathologists who verified that the estimated tumor cell content was above 30%.

### Treatments

We performed gross or subtotal tumor removal on 56 patients (52.3%) and partial tumor removal (PTR) or biopsy on 51 patients (47.7%). Additionally, we treated 105 GBM patients with temozolomide during radiotherapy as per the Stupp protocol and also performed subsequent temozolomide maintenance treatments.^12^ However, 3 patients were not treated because of severe clinical conditions, such as advanced age or low Karnofsky Performance Status (KPS) scores.

### DNA Extraction and Quantification

We extracted DNA from the FFPE samples using the Maxwell 16 FFPE Tissue LEV DNA Purification kit (Promega, Madison, WI, USA) according to the manufacturer’s instructions and measured the concentration using the dsDNA BR Assay kit (Life Technologies, Carlsbad, CA, USA) in the Qubit 3.0 Fluorometer. We evaluated DNA quality by diluting the extracted DNA to 5-10 ng/µL and using it as a template for polymerase chain reaction (PCR). We conducted PCR using the QIAseq DNA QuantiMIZE kit (Qiagen, Hilden, Germany).

### Next-generation Sequencing

We analyzed the extracted DNA by next-generation sequencing (NGS) using an amplicon-based glioma-tailored gene panel as described previously.^13^ Thereafter, we identified the copy number variations (CNVs) and single nucleotide polymorphisms, including those of genes *PDGFRA*, *TERTp*, *CDKN2A/B*, *NF1*, *PTEN*, *RB1*, *TP53*, and *EGFR*. In this regard, amplicon sequences were aligned to the human reference genome GRCh37 (hg19) at the target region. Data were analyzed using the Qiagen web portal service (https://www.qiagen.com/). Based on previous reports,^14,15^ we defined gain as 3-5 copies and amplification as >5 copies.

### Multiplex Ligation-dependent Probe Amplification

CNVs were validated using a MLPA kit (P105-2; MRC-Holland, Amsterdam, the Netherlands) containing *PDGFRA*-specific probes, with 6 other probes used as control probes (http://www.mlpa.com), in accordance with the manufacturer’s protocol.^16^ Denatured fragments were separated and quantified by electrophoresis using an ABI 3730 capillary sequencer (Applied Biosystems, Nieuwerkerk aan den IJssel, the Netherlands) and analyzed using GeneMapper (Applied Biosystems) and Coffalyser software (MRC-Holland). Based on previous publications, the CNV category was classified using the following thresholds: gain (1.2 ≤ × < 2.0), amplification (× ≥ 2.0).^17^

### Methylation-specific Polymerase Chain Reaction

We performed bisulfite modification of the extracted genomic DNA using the EpiTect Bisulfite Kit (Qiagen). After the conversion, genomic DNA was amplified for the target *O*^6^-methylguanine-DNA methyltransferase promoter (*MGMTp*) region with primers specific to the methylated or unmethylated template using KOD One PCR Master Mix (TOYOBO). For methylation-specific PCR (MSP), 2 pairs of primers specific for either the methylated or the unmethylated *MGMTp* region were used as previously described.^16^ The amplification was performed with an initial denaturation at 98°C for 1 minute and 40 cycles of 98°C for 10 seconds, 64°C for 5 seconds. Analysis was performed using the Shimadzu MCE-202 MultiNA (Shimadzu) on the DNA-1000 kit. The cutoff value for methylation was >4% as previously described.^18^

### Analyses of the Memorial Sloan Kettering Cancer Center data

We retrieved the molecular characteristics of the GBM cohort from previous studies. Subsequently, we analyzed 456 cases from the Memorial Sloan Kettering Cancer Center (MSKCC) cohort, excluding the *H3F3A*, *IDH1/2*, and *BRAF* V600E-mutant cases.^19^ All cases were conclusively diagnosed as having *IDH* wild-type GBM using cBioPortal for Cancer Genomics (https://cbioportal.org).

### Data Analysis

We visualized and analyzed our data using the cBioPortal for Cancer Genomics tools OncoPrinter (cbioportal.org/oncoprinter).^20,21^ Additionally, we analyzed the data using EZR (Saitama Medical Center, Jichi Medical University, Saitama, Japan), a graphical user interface of the R software (The R Foundation for Statistical Computing, Vienna, Austria). We compared the risk groups and the patient characteristics using the chi-square (χ ^2^) test and the Kaplan-Meier log-rank test, respectively. We also performed univariate and multivariate Cox regression analyses. A difference was considered statistically significant at *P* < .05.

## Results

### Frequency of *PDGFRA* Gain/Amplification and *MGMTp* Methylation Status in *IDH* Wild-type GBM

A total of 107 cases of *IDH* wild-type GBM were examined in this study. The median overall survival (OS) was 19.7 months. Using NGS, *PDGFRA* copy number was assessed in all cases; it was between 1 and 3 in 76 cases (71.0%), between 3 and 5 in 11 cases (10.3%), and >5 in 20 cases (18.7%). We determined 3-5 copies as *PDGFRA* gain, and >5 copies as *PDGFRA* amplification. To validate *PDGFRA* gene gain/amplification performed by our NGS panel, we conducted MLPA on 15 selective GBM cases, comprising 3 defined by NGS as showing *PDGFRA* gain (copy number range: 3-5), 5 defined by NGS as showing *PDGFRA* amplification (copy number range >5), and 7 defined by NGS as lacking *PDGFRA* gain/amplification (copy number range: 1-3). The CNVs of our NGS analysis were consistent with those of the MLPA analysis except for 1 case (Supplementary Table 1). Moreover, to determine the cutoff value of *PDGFRA* CNVs corresponding to the minimal *P* values of log-rank test for OS, we investigated and plotted the *P* values depending on the various *PDGFRA* copy numbers (range 1-10 copies) as threshold point (Supplementary Figure 1). We determined >3 *PDGFRA* copies as the optimal cutoff value because *P* values get minimal when threshold copy number is 3. Consequently, we detected *PDGFRA* gain in 11 tumor samples (10.3%) and *PDGFRA* amplification in 20 tumor samples (18.7%); the remaining 76 samples (71.0%) harbored no *PDGFRA* gain/amplification.

*MGMTp* methylation analysis using MSP was assessed in all cases, including 12 cases as demonstrated in Supplementary Figure 2. Consequently, we detected *MGMTp* methylation in 58 tumor samples (54.2%); in the remaining 49 samples (45.8%), *MGMTp* methylation was not observed.

### Genetic and Clinical Factors Influencing Prognosis

First, we analyzed whether the identified genetic markers were prognostic markers. Notably, *PDGFRA* gain/amplification and unmethylated *MGMTp* were significant predictors of poor prognosis, as determined by our univariate [HR: 2.22 (1.30-3.78), *P* = .003; and HR: 2.10 (1.24-3.57), *P* = .006, respectively] and multivariate analyses [HR: 2.52 (1.34-4.76), *P* = .004; and HR: 2.28 (1.28-4.07), *P* = .005, respectively; Table 1].

**Table 1.** Genetic Prognostic Factors

	Univariate Analysis		Multivariate Analysis	
Genetic Marker	HR (95% CI)	*P* value	HR (95% CI)	*P* value
*CDKN2A/B* homdel	1.24 (0.74-2.10)	.417	1.26 (0.69-2.37)	.442
*EGFR* amp	0.82 (0.48-1.40)	.462	0.89 (0.46-1.70)	.721
*PTEN* loss and/or mut	0.72 (0.39-1.33)	.294	0.58 (0.27-1.24)	.158
*TP53* loss and/or mut	1.08 (0.64-1.82)	.782	1.04 (0.59-1.82)	.902
*TERTp* mut	1.16 (0.67-2.00)	.599	1.86 (0.87-3.96)	.110
Unmethylated *MGMTp*	2.10 (1.24-3.57)	.006*	2.28 (1.28-4.07)	.005*
*PDGFRA* gain/amp	2.22 (1.30-3.78)	.003*	2.52 (1.34-4.76)	.004*

amp, amplification; homdel, homozygous deletion; mut, mutation.

The symbol * indicates statistical significance.

Second, we identified the clinical prognostic factors, which included analysis of the genetic markers for *PDGFRA* gain/amplification and unmethylated *MGMTp*. Our univariate analysis revealed that age [HR: 2.53 (1.47-4.33), *P* < .001], PTR/biopsy [HR: 2.05 (1.20-3.48), *P* = .008], unmethylated *MGMTp* [HR: 2.10 (1.24-3.57), *P* = .006], and *PDGFRA* gain/amplification [HR: 2.22 (1.30-3.78), *P* = .003] were significantly associated with poor prognosis (Table 2). Thereafter, we adjusted the covariates, including sex and age and KPS score and the extent of tumor resection, in the multivariate Cox proportional hazards model. This analysis corroborated the finding that *PDGFRA* gain/amplification and unmethylated *MGMTp* were independent prognostic markers of OS in patients with *IDH* wild-type GBM [HR: 1.82 (1.00-3.31), *P* = .049; and HR: 3.00 (1.67-5.39), *P* < .001, respectively; Table 2].

**Table 2.** Clinical and Genetic Prognostic Factors

	Univariate Analysis		Multivariate Analysis	
Prognostic Factor	HR (95% CI)	*P* value	HR (95% CI)	*P* value
Sex (male)	1.60 (0.93-2.75)	.093	1.60 (0.91-2.83)	.104
Age (>70 years)	2.53 (1.47-4.33)	<.001*	2.72 (1.51-4.92)	<.001*
KPS score (≤80 points)	1.46 (0.81-2.64)	.210	1.76 (0.92-3.37)	.088
PTR/biopsy	2.05 (1.20-3.48)	.008*	1.85 (1.03-3.32)	.041*
Unmethylated *MGMTp*	2.10 (1.24-3.57)	.006*	3.00 (1.67-5.39)	<.001*
*PDGFRA* gain/amp	2.22 (1.30-3.78)	.003*	1.82 (1.00-3.31)	.049*

KPS, Karnofsky Performance Status; PTR, partial tumor removal.

The symbol * indicates statistical significance.

### Genetic and Clinical Factors Associated With and Without *PDGFRA* Gain/Amplification

Table 3 compares the genetic and clinical factors of the patients based on their *PDGFRA* status. We discovered that the *TP53* mutation was more common in GBMs with *PDGFRA* gain/amplification than in those without (*P* = .011). Conversely, alterations in *EGFR* (*P* < .001), and *TERTp* (*P* < .001) were more common in GBMs without *PDGFRA* gain/amplification than in those with the gain/amplification (Supplementary Figure 3). In our study, no case had both *PDGFRA* amplification and *EGFR* amplification (Supplementary Figure 3). Furthermore, patients with GBM with *PDGFRA* gain/amplification were associated with higher age (*P* = .031), higher Ki-67 score (*P* = .019), and lower extent of surgical resection (*P* = .033) than those without *PDGFRA* gain/amplification.

**Table 3.** Background of Patients With and Without *PDGFRA* Gain/Amplification

Prognostic Factor		All (n = 107)	*PDGFRA* Gain/Amp (n = 31)	*PDGFRA* Intact (n = 76)	*P* value
Sex	Male	61 (57.0%)	19 (61.3%)	42 (55.3%)	.668
	Female	46 (43.0%)	12 (38.7%)	34 (44.7%)	
Age	<70 years	60 (56.1%)	12 (38.7%)	48 (63.2%)	.031*
	>70 years	47 (43.9%)	19 (61.3%)	28 (36.8%)	
KPS score	>80 points	31 (29.0%)	10 (32.3%)	21 (27.6%)	.645
	≤80 points	76 (71.0%)	21 (67.7%)	55 (72.4%)	
Resection	GTR/STR	56 (52.3%)	11 (35.5%)	45 (59.2%)	.033*
	PTR/biopsy	51 (47.7%)	20 (64.5%)	31 (40.8%)	
Ki-67	>35%	56 (52.3%)	22 (71.0%)	34 (44.7%)	.019*
	<35%	51 (47.7%)	9 (29.0%)	42 (55.3%)	
*CDKN2A/B* homdel		51 (47.7%)	16 (51.6%)	35 (46.1%)	.672
*NF1* loss and/or mut		23 (21.5%)	4 (12.9%)	19 (25.0%)	.202
*PTEN* loss and/or mut		73 (68.2%)	17 (54.8%)	56 (73.7%)	.069
*RB1* loss and/or mut		39 (36.4%)	10 (32.3%)	29 (38.2%)	.660
*TERTp* mut		70 (65.4%)	12 (38.7%)	58 (76.3%)	<.001*
*TP53* loss and/or mut		52 (48.6%)	21 (67.7%)	31 (40.8%)	.011*
*EGFR* amp		43 (40.2%)	2 (6.5%)	41 (53.9%)	<.001*
Unmethylated *MGMTp*		49 (45.8%)	13 (41.9%)	36 (47.4%)	.672

amp, amplification; GTR, gross tumor removal; homdel, homozygous deletion; KPS, Karnofsky Performance Status; mut, mutation; PTR, partial tumor removal; STR, subtotal tumor removal.

The symbol * indicates statistical significance.

### *PDGFRA* Gain/Amplification and Unmethylated *MGMTp* Are Associated With Poor Patient Prognoses

**Figure 1.**
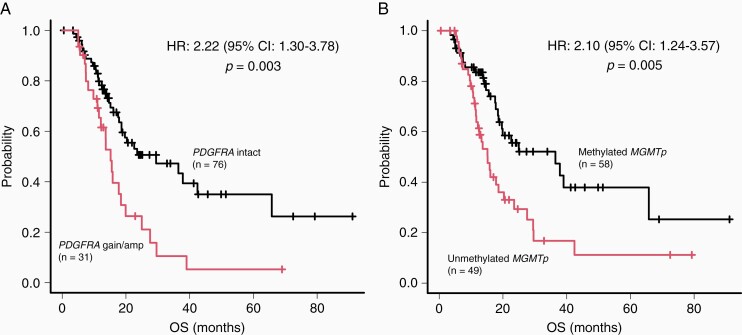
Prognostic impact of *PDGFRA* and *MGMTp* status. (A) *IDH* wild-type glioblastoma (GBM) cases with *PDGFRA* gain/amplification exhibiting significantly shorter overall survival (OS) than those without *PDGFRA* gain/amplification. (B) *IDH* wild-type GBM cases with unmethylated *MGMTp* exhibiting significantly shorter OS than those with methylated *MGMTp*.

**Figure 2.**
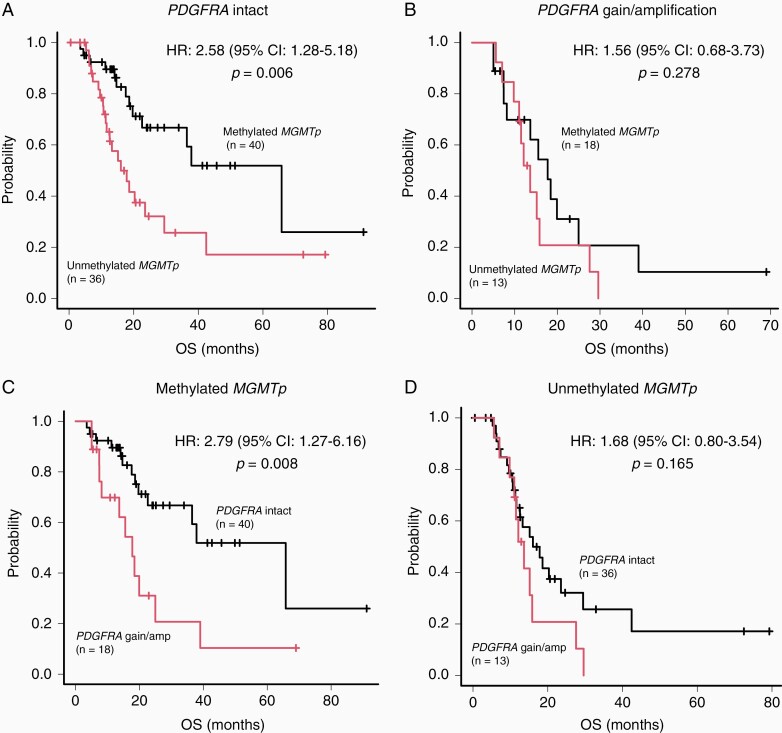
Survival analysis of *IDH* wild-type glioblastoma (GBM) cases according to *PDGFRA* and *MGMTp* status. (A) Survival analysis of patients with *IDH* wild-type GBM harboring either unmethylated or methylated *MGMTp* without *PDGFRA* gain/amplification. (B) Survival analysis of patients with *IDH* wild-type GBM harboring either unmethylated or methylated *MGMTp* with *PDGFRA* gain/amplification. (C) Kaplan-Meier analysis for OS of patients with and without *PDGFRA* gain/amplification in *IDH* wild-type GBMs with methylated *MGMTp*. (D) Kaplan-Meier survival analysis of patients with and without *PDGFRA* gain/amplification in *IDH* wild-type GBMs with unmethylated *MGMTp*.

We observed a significant difference in the median OS of patients with and without *PDGFRA* gain/amplification (15.2 and 29.5 months, respectively; *P* = .003; Figure 1A). Subsequently, we validated this observation by analyzing the data of 456 patients with *IDH* wild-type GBM obtained from the MSKCC cohort. We observed a significant difference in the median OS of the patients with and without *PDGFRA* amplification (16.6 and 23.5 months, respectively; *P* = .017; Supplementary Figure 4). Moreover, unmethylated *MGMTp* was a significant predictor of poor prognosis (*P* = .005; Figure 1B). In patients with *PDGFRA* intact, unmethylated *MGMTp* was a significant predictor of poor prognosis (*P* = .006, Figure 2A), whereas in patients with *PDGFRA* gain/amplification, there was no significant difference between the median OS of patients with methylated *MGMTp* and those with unmethylated *MGMTp* (*P* = .278, Figure 2B). In addition, in patients with methylated *MGMTp*, *PDGFRA* gain/amplification was a significant predictor of poor prognosis (*P* = .008, Figure 2C), whereas in patients with unmethylated *MGMTp*, there was no significant difference between the median OS of patients with and without *PDGFRA* gain/amplification (*P* = .165, Figure 2D).

### Prognostic Impact of the Combination of *PDGFRA* Gain/Amplification and *MGMTp* Methylation Status in *IDH* Wild-type GBM

**Figure 3.**
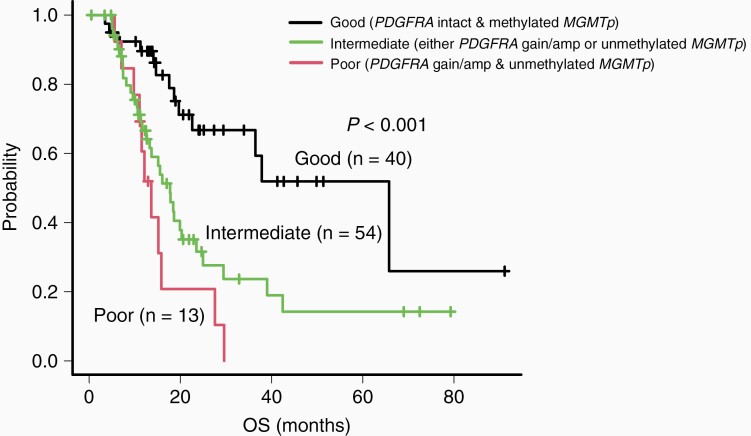
Risk stratification of *IDH* wild-type glioblastoma cases based on *PDGFRA* and *MGMTp* mutational status. The Kaplan-Meier overall survival curves as per risk stratification. The poor-, intermediate-, and good-prognosis groups include patients with *PDGFRA* gain/amplification and unmethylated *MGMTp*, with either *PDGFRA* gain/amplification or unmethylated *MGMTp*, and with *PDGFRA* intact and methylated *MGMTp*, respectively.

We aimed to perform a risk stratification of *IDH* wild-type GBM. *PDGFRA* gain/amplification and unmethylated *MGMTp* were associated with clinical outcomes of the patients and were independent prognostic factors in the multivariate analysis. Therefore, we included these 2 molecular markers for the subsequent risk stratification. We subdivided *IDH* wild-type GBM into 3 groups according to the status of *PDGFRA* and *MGMTp* (Figure 3). *IDH* wild-type patients with GBM with *PDGFRA* gain/amplification and unmethylated *MGMTp* were assigned to the poor-prognosis group, whereas those harboring either *PDGFRA* gain/amplification or unmethylated *MGMTp* were assigned to the intermediate-prognosis group. Lastly, patients with *PDGFRA* intact and methylated *MGMTp* were assigned to the good-prognosis group. Remarkably, our Kaplan-Meier survival analysis indicated that these groups were correlated with the OS of the patients (*P* < .001; Figure 3).

However, these groups were not associated with any clinical factors, including sex, age, the extent of surgical resection, KPS score, Ki-67 score, and other genetic factors, except for *EGFR* amplification (Supplementary Table 2).

## Discussion

In this study, we demonstrated the impact of *PDGFRA* gain/amplification as a prognostic marker of *IDH* wild-type GBM, along with the *MGMTp* methylation status. Studies have detected *PDGFRA* amplification in approximately 8.5%-29% of GBM cases^9,22–27^; our results corroborated these observations. We demonstrated that *PDGFRA* gain/amplification is a significant predictor of poor prognoses in patients with *IDH* wild-type GBM; this was validated using the MSKCC dataset. Consistent with previous reports,^28–30^ we also identified the age and extent of surgical resection and unmethylated *MGMTp* as independent GBM prognostic factors, highlighting the accuracy of our study. *PDGFRA* gain/amplification and unmethylated *MGMTp* were identified as independent prognostic markers in multivariate analysis; however, the hazard ratio and *P* value for *PDGFRA* gain/amplification became less significant than those for unmethylated *MGMTp* when clinical factors were included. This could be attributed to the fact that *PDGFRA* gain/amplification is associated with higher patient age and lower extent of resection. Therefore, *PDGFRA* gain/amplification as a prognostic factor is confounded by age and extent of resection. To date, the prognostic value of *PDGFRA* gain/amplification in GBM remains controversial. While some previous studies have reported no prognostic impact of *PDGFRA* amplification in GBM,^23,26,27,31^ one study has reported poor prognostic impact of this amplification in GBM.^32^ These differences might be attributed to the different methods used to detect gene amplification, or intratumor heterogeneity, a notable feature of GBM.^33^ Studies have demonstrated that NGS panel-based identification of CNVs is more sensitive than conventional methods, including multiplex ligation-dependent probe amplification (MLPA) and PCR.^34^ The MLPA method renders false-negative results because of contamination by unamplified non-neoplastic and neoplastic DNA caused by intratumoral heterogeneity.^35^ To solve these problems, we histologically evaluated all the tissue samples by board-certified pathologists and measured the estimated tumor cell content. Following this, we extracted the tumor cell DNA, highlighting highly reliable data. In contrast, studies have linked *PDGFRA* amplification with the significantly worse OS of patients with *IDH*-mutant GBM^8^ and WHO grade II and III tumors,^36,37^ which is different from the results of the patient group analyzed in this study. Moreover, Alnahhas et al reported that *PDGFRA* amplification was associated with poor survival only in *EGFR/ERBB*-altered GBM.^38^ However, in our study, no case had both *PDGFRA* amplification and *EGFR* amplification, suggesting that our patient background was different from that of the patient group analyzed in their study.

Cui et al reported that *PDGFRA* alterations are associated with the involvement of the corpus callosum, resulting in the low extent of surgical resection values.^9^ Therefore, we assume that *PDGFRA* amplification may be associated with poor prognosis due to the low extent of surgical resection. In our study, *IDH* wild-type GBM with *PDGFRA* gain/amplification was significantly associated with older age, consistent with previous reports. Moreover, it was significantly associated with a higher Ki-67 score. Previous studies have shown that the Ki-67 score is an important prognostic factor in GBM and a marker of cell proliferation.^39–41^ Thus, our results demonstrated that *PDGFRA* gain/amplification causes poor prognoses in patients with *IDH* wild-type GBM by increasing the proliferative ability of tumors and increasing the rate of incomplete resection of tumors.

*MGMTp* methylation is a well-established favorable prognostic marker for survival and predicts the response to temozolomide in patients with GBM.^28,42^ Since *PDGFRA* gain/amplification and unmethylated *MGMTp* were independent prognostic markers, we investigated the potential interaction between *PDGFRA* gain/amplification and unmethylated *MGMTp* in patients with GBM and hypothesized that these markers improved the risk stratification of *IDH* wild-type GBM. Consequently, our finding that the subset of GBMs with *PDGFRA* gain/amplification and unmethylated *MGMTp* have the poorest prognosis and GBMs with *PDGFRA* intact and methylated *MGMTp* have the most favorable prognosis has important clinical implications. We demonstrated that such stratification, surprisingly, is independent of clinical factors, including age, sex, the extent of resection, KPS score, and other genetic factors except for *EGFR* amplification. Our most striking finding was that the prognostic impact of *PDGFRA* gain/amplification is one of the most powerful predictors of survival in patients with GBM, along with the *MGMTp* methylation status. Furthermore, *PDGFRA* gain/amplification in combination with the *MGMTp* methylation status improves individual prognosis in patients with IDH wild-type GBM.

This study has several limitations. First, it was a retrospective study with a small sample size. Second, differences in molecular biology techniques should be considered. We identified CNVs using NGS, whereas other studies have used MLPA, PCR, or fluorescent in situ hybridization for this purpose.^22,23,25–27,36^

## Conclusions

We report that *PDGFRA* gain/amplification is a predictor of poor prognosis in *IDH* wild-type GBM. Our study illustrates the potential use of molecular markers for a refined stratification of *IDH* wild-type GBM. We recommend the incorporation of *PDGFRA* gain/amplification and *MGMTp* in the molecular stratification of *IDH* wild-type GBM. Such a stratification will likely provide precise information to patients and help influence their bedside decisions.

## Supplementary Material

vdac097_suppl_Supplementary_Material

## Data Availability

All data used and analyzed in the current study are available from the corresponding author upon reasonable request.
