# Supplementary material for: Prognostic impact of PDGFRA gain/amplification and MGMT promoter methylation status in patients with IDH wild-type glioblastoma
Source: Neurooncol Adv. 2022 Jun 21;4(1):vdac097. doi: 10.1093/noajnl/vdac097 (PMC9332894; doi:10.1093/noajnl/vdac097)
Supplement: vdac097_suppl_Supplementary_Material [file vdac097_suppl_supplementary_material.zip › Supplementary table 2.docx]

Table S2. Clinical features of IDH wildtype GBMs according to risk groups

| Prognostic factor | | Poor (n=13) | Intermediate (n=54) | Good (n=40) | *p*-value |
| --- | --- | --- | --- | --- | --- |
| Sex | male | 11 (84.6%) | 29 (53.7%) | 21 (52.5%) | 0.100 |
|  | female | 2 (15.4%) | 25 (46.3%) | 19 (47.5%) |  |
| Age | | 68.7±9.1 | 65.8±14.8 | 68.1±10.2 | 0.601 |
| KPS score | >80 points | 5 (38.5%) | 17 (31.5%) | 9 (22.5%) | 0.431 |
|  | ≤80 points | 8 (61.5%) | 37 (68.5%) | 31 (77.5%) |  |
| Resection | GTR/STR | 5 (38.5%) | 29 (53.7%) | 22 (55.0%) | 0.605 |
|  | PTR/biopsy | 8 (61.5%) | 25 (46.3%) | 18 (45.0%) |  |
| Ki-67 | >35% | 7 (53.8%) | 28 (51.9%) | 21 (52.5%) | 1.000 |
|  | <35% | 6 (46.2%) | 26 (48.1%) | 19 (47.5%) |  |
| *CDKN2A/B* homdel | | 5 (38.5%) | 25 (46.3%) | 21 (52.5%) | 0.655 |
| *NF1* loss and/or mut | | 2 (15.4%) | 11 (20.4%) | 10 (25.0%) | 0.797 |
| *PTEN* loss and/or mut | | 10 (76.9%) | 34 (63.0%) | 29 (72.5%) | 0.560 |
| *RB1* loss and/or mut | | 5 (38.5%) | 20 (37.0%) | 14 (35.0%) | 1.000 |
| *TERTp* mut | | 7 (53.8%) | 35 (64.8%) | 28 (70.0%) | 0.554 |
| *TP53* loss and/or mut | | 8 (61.5%) | 29 (53.7%) | 15 (37.5%) | 0.181 |
| *EGFR* amp | | 2 (15.4%) | 18 (33.3%) | 23 (57.5%) | 0.009* |

KPS, Karnofsky Performance Status; GTR, gross tumor removal; STR, subtotal tumor removal; PTR, partial tumor removal; mut, mutation; amp, amplification; homdel, homozygous deletion

*indicates statistical significance.
