# Supplementary material for: Prognostic impact of PDGFRA gain/amplification and MGMT promoter methylation status in patients with IDH wild-type glioblastoma
Source: Neurooncol Adv. 2022 Jun 21;4(1):vdac097. doi: 10.1093/noajnl/vdac097 (PMC9332894; doi:10.1093/noajnl/vdac097)
Supplement: vdac097_suppl_Supplementary_Material [file vdac097_suppl_supplementary_material.zip › Supplementary figure 1.pptx]

## Slide 1
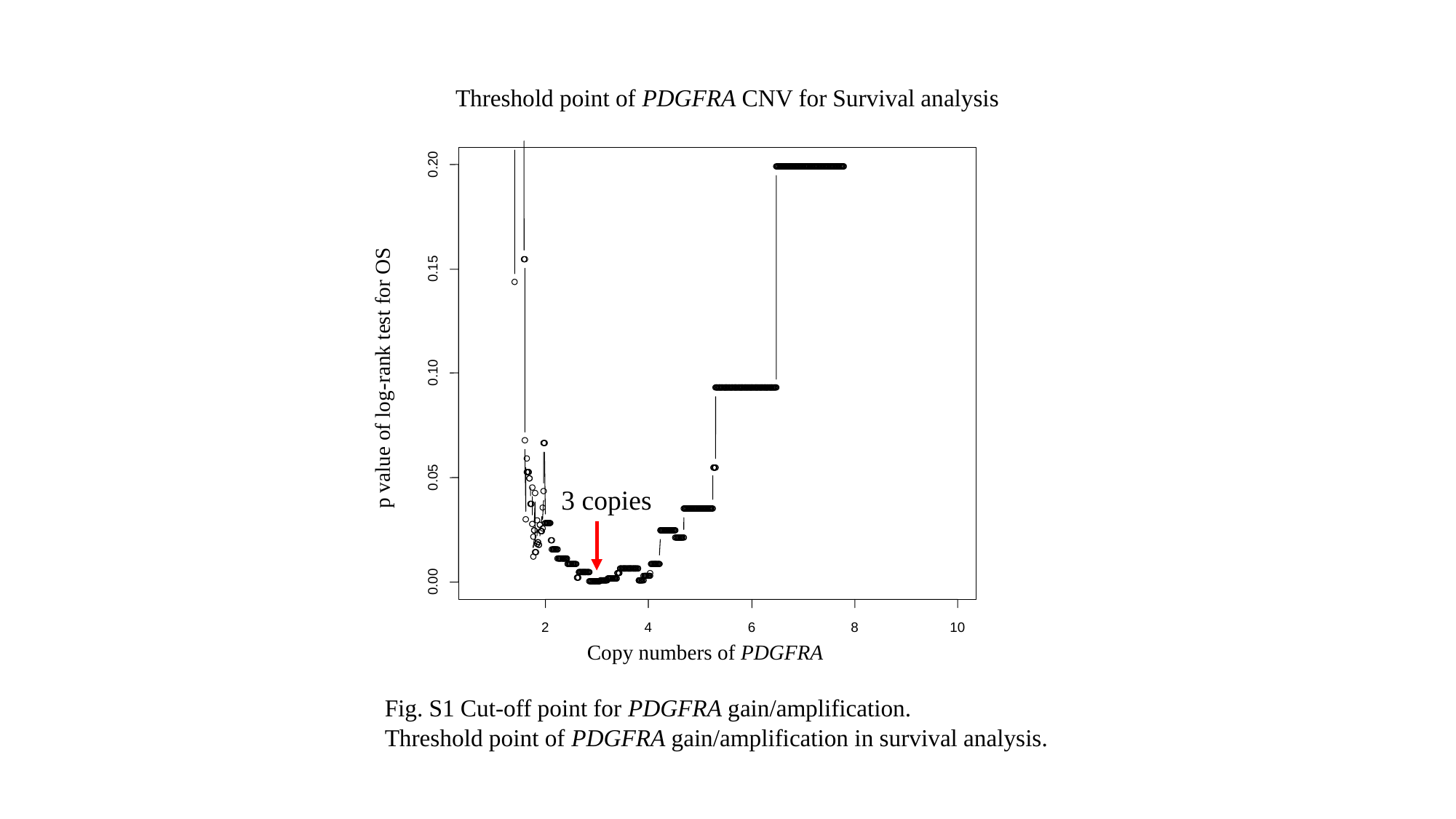

Threshold point of PDGFRA CNV for Survival analysis
0.20
0.15
0.10
p value of log-rank test for OS
0.05
0.00
2
4
6
8
10
Copy numbers of PDGFRA
3 copies
Fig. S1 Cut-off point for PDGFRA gain/amplification.
Threshold point of PDGFRA gain/amplification in survival analysis.
