# Supplementary material for: Prognostic impact of PDGFRA gain/amplification and MGMT promoter methylation status in patients with IDH wild-type glioblastoma
Source: Neurooncol Adv. 2022 Jun 21;4(1):vdac097. doi: 10.1093/noajnl/vdac097 (PMC9332894; doi:10.1093/noajnl/vdac097)
Supplement: vdac097_suppl_Supplementary_Material [file vdac097_suppl_supplementary_material.zip › Supplementary figure 2.pptx]

## Slide 1
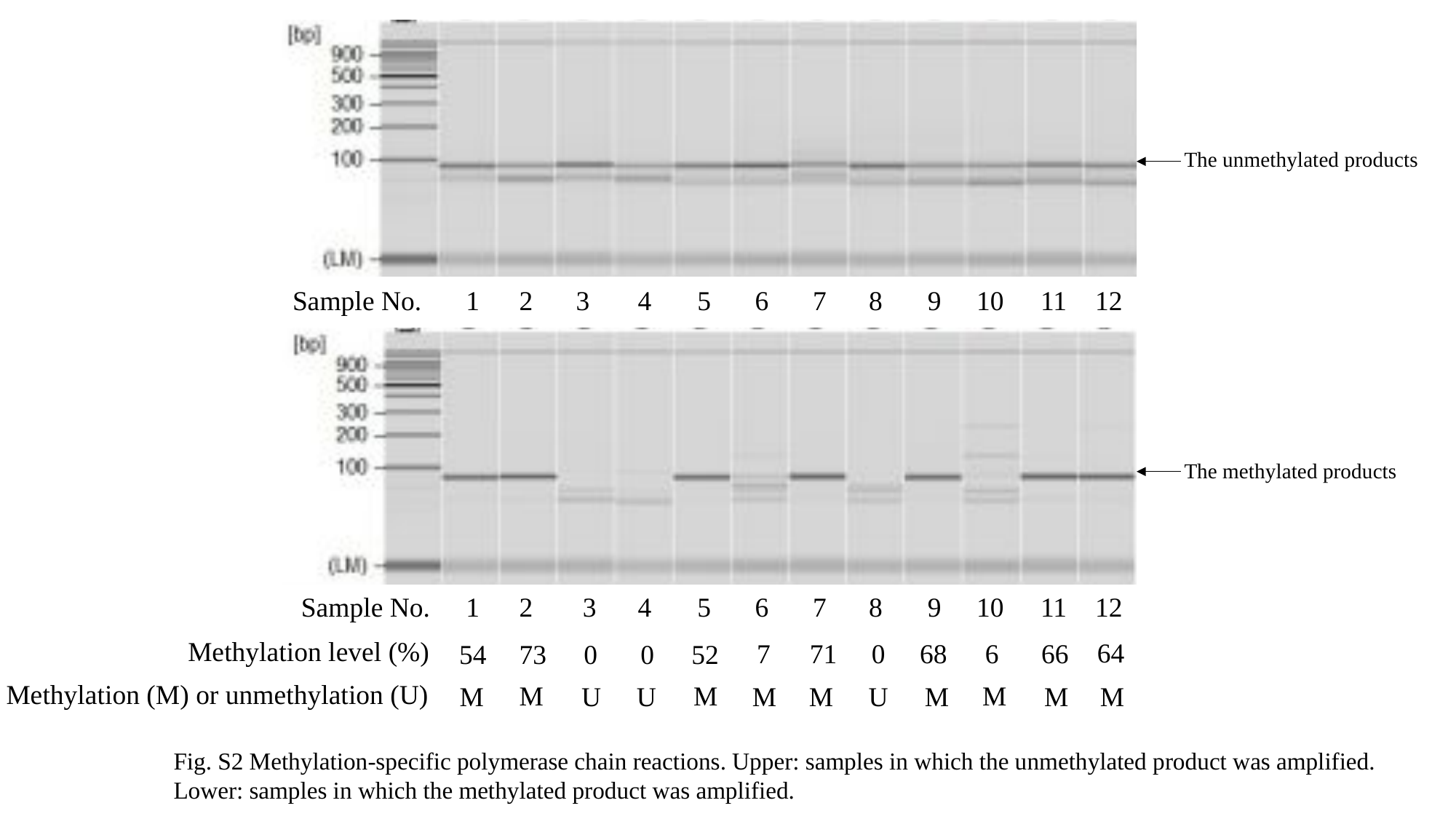

The unmethylated products
Sample No.
1
3
4
5
6
7
8
9
10
11
12
2
The methylated products
Sample No.
1
3
4
5
6
7
8
9
10
11
12
2
Methylation level (%)
64
68
71
66
0
6
7
54
73
52
0
0
Methylation (M) or unmethylation (U)
M
M
M
M
M
M
U
M
M
U
M
U
Fig. S2 Methylation-specific polymerase chain reactions. Upper: samples in which the unmethylated product was amplified. Lower: samples in which the methylated product was amplified.
