# Supplementary material for: Prognostic impact of PDGFRA gain/amplification and MGMT promoter methylation status in patients with IDH wild-type glioblastoma
Source: Neurooncol Adv. 2022 Jun 21;4(1):vdac097. doi: 10.1093/noajnl/vdac097 (PMC9332894; doi:10.1093/noajnl/vdac097)
Supplement: vdac097_suppl_Supplementary_Material [file vdac097_suppl_supplementary_material.zip › supplementary figure 3.pptx]

## Slide 1
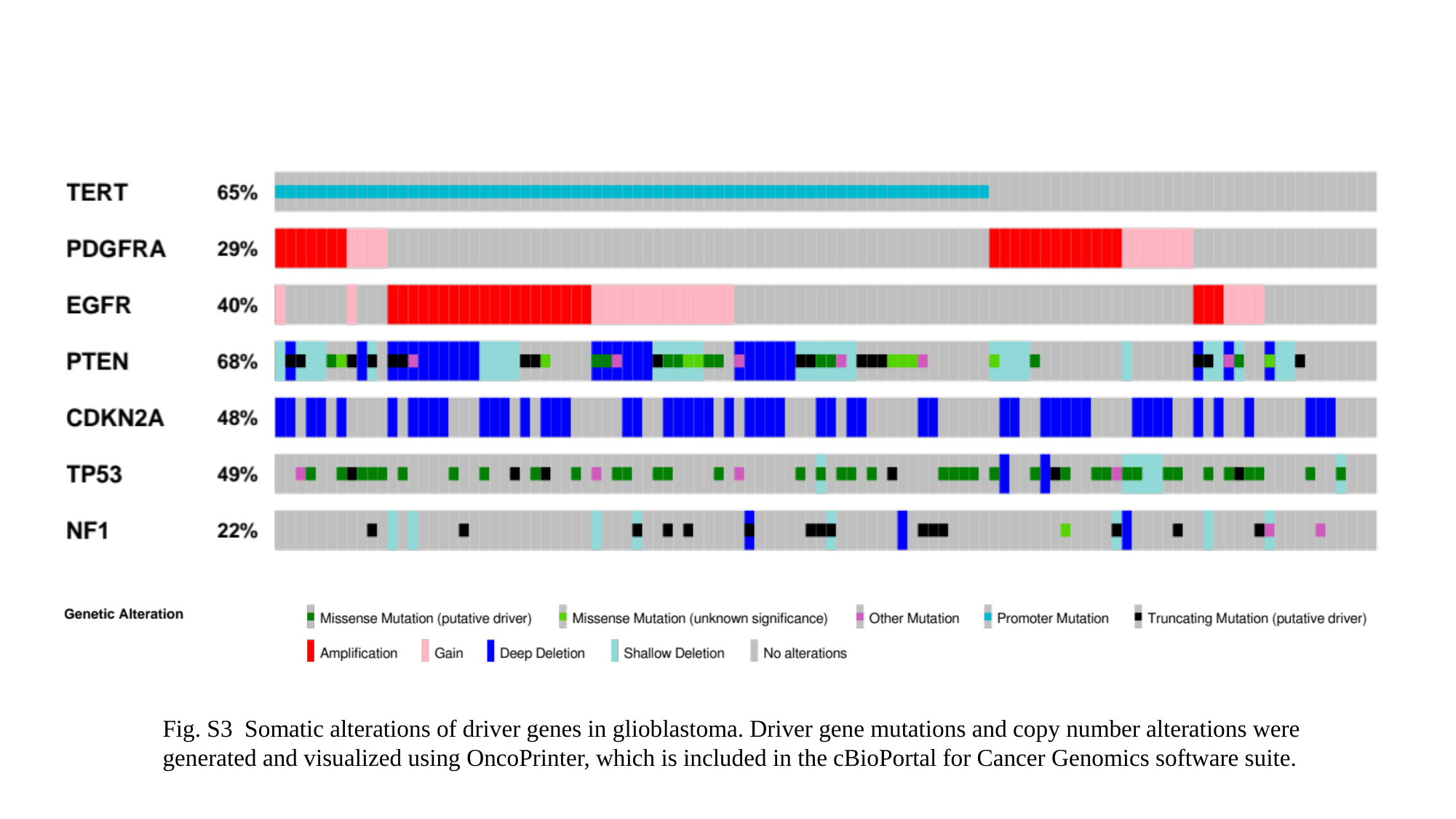

Fig. S3 Somatic alterations of driver genes in glioblastoma. Driver gene mutations and copy number alterations were generated and visualized using OncoPrinter, which is included in the cBioPortal for Cancer Genomics software suite.
