# Supplementary material for: Prognostic impact of PDGFRA gain/amplification and MGMT promoter methylation status in patients with IDH wild-type glioblastoma
Source: Neurooncol Adv. 2022 Jun 21;4(1):vdac097. doi: 10.1093/noajnl/vdac097 (PMC9332894; doi:10.1093/noajnl/vdac097)
Supplement: vdac097_suppl_Supplementary_Material [file vdac097_suppl_supplementary_material.zip › Supplementary figure 4.pptx]

## Slide 1
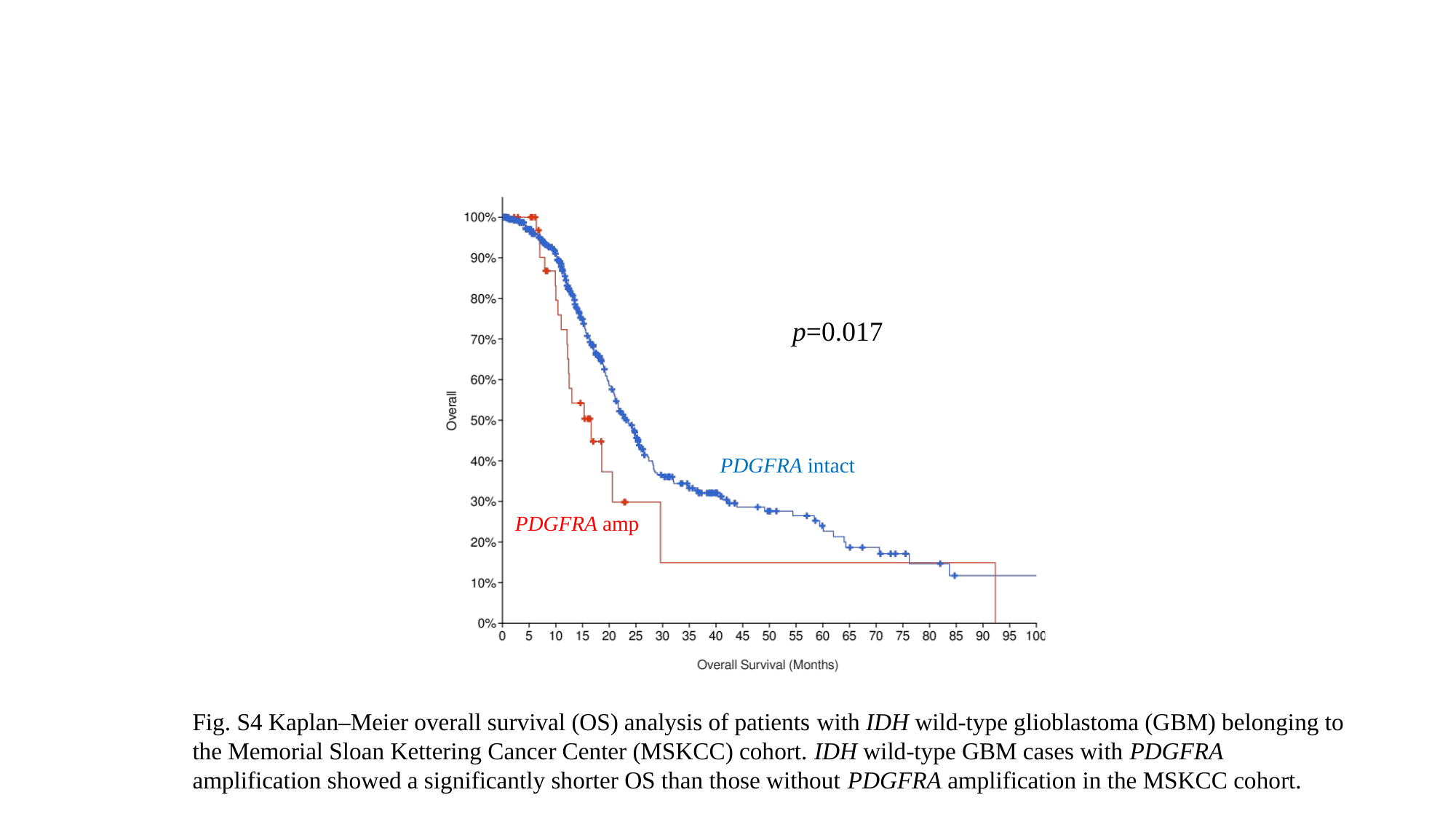

p=0.017
PDGFRA intact
PDGFRA amp
Fig. S4 Kaplan–Meier overall survival (OS) analysis of patients with IDH wild-type glioblastoma (GBM) belonging to the Memorial Sloan Kettering Cancer Center (MSKCC) cohort. IDH wild-type GBM cases with PDGFRA amplification showed a significantly shorter OS than those without PDGFRA amplification in the MSKCC cohort.
