# Supplementary material for: Prognostic impact of PDGFRA gain/amplification and MGMT promoter methylation status in patients with IDH wild-type glioblastoma
Source: Neurooncol Adv. 2022 Jun 21;4(1):vdac097. doi: 10.1093/noajnl/vdac097 (PMC9332894; doi:10.1093/noajnl/vdac097)
Supplement: vdac097_suppl_Supplementary_Material [file vdac097_suppl_supplementary_material.zip › Supplementary table 1.docx]

Table S1. Comparison of NGS analysis and MLPA analysis for detecting CNVs

| Case | Copy numbers by NGS | | MLPA ratio | |
| --- | --- | --- | --- | --- |
| 1 | 63.5 | amplification | 11.2 | amplification |
| 2 | 45.5 | amplification | 18.8 | amplification |
| 3 | 1.5 | intact | 1.0 | intact |
| 4 | 1.7 | intact | 1.0 | intact |
| 5 | 1.4 | intact | 0.9 | intact |
| 6 | 1.7 | intact | 0.9 | intact |
| 7 | 1.5 | intact | 1.0 | intact |
| 8 | 1.3 | intact | 0.9 | intact |
| 9 | 24.8 | amplification | 7.3 | amplification |
| 10 | 1.4 | intact | 1.0 | intact |
| 11 | 5.3 | amplification | 1.4 | gain |
| 12 | 4.6 | gain | 1.9 | gain |
| 13 | 9.5 | amplification | 2.3 | amplification |
| 14 | 3.9 | gain | 1.3 | gain |
| 15 | 4.2 | gain | 1.5 | gain |
